# President’s Address

**Published:** 1895-11

**Authors:** J. Y. Crawford

**Affiliations:** Nashville, Tenn.


### President's Address.

BY J. Y. CRAWFORD, M.D., D.D.S., NASHVILLE, TENN.

Abstract of paper read at American Dental Association, Aug., 1895.

In his address Dr. Crawford recommended that the two public addresses at the memorial meeting at Philadelphia, together with a synopsis of what was said at the banquet, be incorporated into the annual proceedings. He suggested that " the old question of representation of dental surgery in the medical corps of the army and navy demands an earnest and united effort on the part of our profession. . . . . That a committee be appointed to investigate the propriety of some inquiry being made in regard to the condition of the mouth and teeth of an individual before obtaining life insurance. . . . . Knowing, as we do, how destructive the diseases of the mouth and teeth are to human comfort, happiness and health, it is passingly strange that no attention has been paid to the subject in conducting a medical examination of an applicant for life insurance."

Regarding dental prophylaxis in the scholastic part of our population, he said: " Our system of education is sacrificing the teeth and health of our people, by putting the child in school at too tender an age. . . . . The child should not be put to hard study while he is undergoing the process of tooth-shedding and tooth eruption." He suggested that the association ask medical colleges throughout the country to institute special chairs upon the subject of Dental and Oral Surgery, so that medical students will be required to know something of the diseases of the mouth and teeth and the deleterious influence they have in the impairment of the general health.

In his address Dr. Crawford referred to the question of ethics in no uncertain terms. He spoke of the pernicious use of the secular press advertising patent medicines to the world, and of the regretable mistake of some ministers, professors of universities, etc., giving personal testimonials recommending some nostrum the contents of which they know nothing. Further he said: "If the dental surgeons of the United States would unite in demanding the enactment of such laws as would require of all men, proposing to engage in the practice of dentistry, an observance of ethical conduct, it would be but a short time before the statute books of all the States would be graced with laws that would annihilate the quack and the mountebank."

He requested, also, that all reputable dental colleges formulate a uniform oath or obligation to which the student should subscribe.

